# Supplementary material for: Comparing the outputs of intramural and extramural grants funded by National Institutes of Health
Source: eLife. 2026 Jul 9;14:RP108929. doi: 10.7554/eLife.108929 (PMC13349378; doi:10.7554/eLife.108929)
Supplement: Peer review history

# Editor report: eLife Assessment

**DOI:** 10.7554/eLife.108929.3.sa0

**Authors:** Peter Rodgers

**Affiliations:** eLife United Kingdom

**Keywords:** Important, Solid

This **important** study used five metrics to compare the cost-effectiveness of intramural and extramural research funded by the National Institutes of Health in the United States between 2009 and 2019. They found that each type of research had its own set of strengths: extramural research was more cost-effective in terms of publications, whereas intramural research was more cost-effective in terms of influencing clinical work. The evidence supporting these findings is **solid**.

# Referee report: Reviewer #1 (Public review)

**DOI:** 10.7554/eLife.108929.3.sa1

Summary:

This paper carefully compares intramural vs. extramural National Institutes of Health funded research during 2009-2019, according to a variety of bibliometric indices. They find that extramural awards more cost-effectively fund outputs commonly used for academic review such as number of publications and citations per dollar, while intramural awards are more cost-effective at generating work that influences future clinical work, more closely in line with agency health goals.

Strengths:

Great care was taken in selecting and cleaning the data, and in making sure that intramural vs. extramural projects were compared appropriately. The data has statistical validation. The trends are clear and convincing.

# Referee report: Reviewer #2 (Public review)

**DOI:** 10.7554/eLife.108929.3.sa2

This article reports a cost-effectiveness comparison of intramural and extramural that NIH funded between 2009 and 2019. Using data obtained from NIH RePORTER, they linked total project costs to publication output, using robust validated metrics including Relative Citation Ratio (RCR), Approximate Potential to Translate (APT), and clinical citations. They find that after adjusting for confounders in regression and propensity-score analyses, extramural projects were generally more cost-effective, though intramural projects were more cost effective for generating clinical citations. They also describe differences in the topics of intramural- and extramural-funded publications, with intramural projects more likely to generate papers on viral infections and immunity or cancer metastases and survival, but less likely to generate papers on pregnancy and maternal health, brain connectivity and tasks, and adolescent experiences and depression. The authors aptly describe the different natures of the intramural and extramural funding models, including that extramural researchers spend much time writing grant applications and that the work described in extramural publications often receives funding from sources other than NIH grants.

Strengths:

The authors leveraged publicly available data (including RePORTER and the iCite repository) and used robust validated metrics (RCR, APT, clinical citations). They carefully considered a large number of confounders, including those related to the PI, and performed several well-described regression analyses.

# Referee report: Reviewer #3 (Public review)

**DOI:** 10.7554/eLife.108929.3.sa3

This article demonstrates a comparative study on two funding mechanisms adopted by the National Institutes of Health (NIH). The authors adopted a quantitative approach and introduced five metrics to compare the output of intramural and extramural grants. These findings reveal the impacts of intramural and extramural grants on the scientific community, providing funders with insights into the future decisions of funding mechanisms they should take.

Strengths:

The authors clearly presented their methods for processing the NIH project data and classifying projects into either intramural or extramural categories. The limitations of the study are also well-addressed.

# Author comment: Author response

**DOI:** 10.7554/eLife.108929.3.sa4

**Authors:** Xiang Zheng, Qiyao Yang, Jai Potnuri, Chaoqun Ni, B Ian Hutchins

**Affiliations:** University of Wisconsin-Madison Madison United States; University of Wisconsin-Madison Madison United States; University of Wisconsin-Madison Madison United States; University of Wisconsin-Madison Madison United States; University of Wisconsin-Madison Madison United States

The following is the authors’ response to the original reviews.

**Public Reviews:**

**Reviewer #1 (Public review):**

Strengths:

Great care was taken in selecting and cleaning the data, and in making sure that intramural vs. extramural projects were compared appropriately. The data has statistical validation. The trends are clear and convincing.

We thank the reviewer for highlighting the strengths of the manuscript.

Weaknesses:

The Discussion is too short and descriptive, and needs more perspective - why are the findings important and what do they mean? Without recommending policy, at least these should discuss possible implications for policy.

The Discussion has been substantially expanded. We added several new paragraphs discussing: the 2024 Senate HELP Committee proposal for NIH reform; implications for portfolio management (positioning extramural for basic research, intramural for clinical translation); generalizability to other agencies (DoD, NSF FFRDCs, DoE national labs); and the extramural program's role in workforce training as a societal benefit distinct from research outputs.

The biggest problem I have with this submission is Figure 3, which shows a big decrease in clinical-related parameters between 2014 and 2019 in both intramural and extramural research (panels C, D and E). There is no obvious explanation for this and I did not see any discussion of this trend, but it cries out for investigation. This might, for example, reflect global changes in funding policies which might also influence the observed closing gaps between intramural and extramural research.

We added an explicit explanation in the Results: because the dataset is truncated at 2020, clinical citations naturally approach zero near the window's end, consistent with the ~7-year lag for clinical citations to accrue documented in prior work (Hutchins et al., 2019). The APT metric declines less steeply because it uses the forward citation network for predictions.

**Reviewer #2 (Public review):**

Strengths:

The authors leveraged publicly available data (including RePORTER and the iCite repository) and used robust validated metrics (RCR, APT, clinical citations). They carefully considered a large number of confounders, including those related to the PI, and performed several well-described regression analyses.

We thank the reviewer for highlighting these strengths of the manuscript

Figure 3A shows intramural projects producing about 2.75 papers per year in 2009, whereas extramural projects are producing just over 1 paper per year. Extramural projects appear to catch up over the next five years. While the authors attempt to explain the difference in their figure legend, another explanation is that the intramural projects started well before 2009 but, as the authors state, intramural data only became available in 2009.

We added a methodological note acknowledging that some intramural projects may have had start dates prior to 2009 that are not captured in the data, and that the ramp-up of new intramural projects is slower because they are more tied to new PI hiring. We also note the exclusion of projects matched in 2008 as possible continuations. However, the slow ramp-up of Intramural costs in Supplemental Figure 3 is consistent with hiring-associated lagged investment suggesting that our filtering of continuing projects was very successful. Nevertheless, because we cannot completely rule out some continuing projects made it through despite our efforts, we have made the caveats mentioned above in the “Comparison of research topics” section of the Results and the Data section of the Methods.

As the authors note, funding information is often complex and difficult to characterize for an analysis like this. How did the authors handle: (i) publications linked to multiple extramural grants; (ii) publications linked to intramural and extramural grants; (iii) publications linked NIH grants and non-NIH grants?

I would think it necessary to somehow apportion credit, as otherwise it would appear that extramural projects are more productive than they truly are.

We have now explicitly stated that papers with both intramural and extramural funding links were excluded, while papers with multiple links within the same funding type were retained. A new Supplemental Figure 6 was added showing the distribution of papers by number of funding sources for both extramural and intramural grants, demonstrating that the vast majority acknowledged only one project. These changes are in the Methods, Data section and Supplemental Figure 6

Apportioning credit among a many-to-many graph like the ones used here is indeed a high value problem to solve, but one with many researcher-degrees-of-freedom about analytical design decisions that impact the results. We are working on a rigorous methodology for this, but the amount of time required to do this well is its own research project, and out of scope for manuscript revisions.

Also, it is not clear if the authors took account of the indirect costs paid by the NIH to universities that have received extramural grants.

We added explicit language clarifying that all cost comparisons use inflation-adjusted total costs (direct + indirect) for extramural grants. We also added a new sensitivity analysis (Supplemental Figure 4) inflating extramural indirect costs by 30% to approximate unrecovered university expenditures, with the finding that the fundamental pattern holds even under this adjustment. These are found in the “Comparison of funding” and “Comparison of cost effectiveness” sections of the Results, as well as Supplemental Figure 4.

**Reviewer #3 (Public review):**

Strengths:

The authors clearly presented their methods for processing the NIH project data and classifying projects into either intramural or extramural categories. The limitations of the study are also well-addressed.

We thank the reviewer for highlighting these strengths of the manuscript

Weaknesses:

The article would benefit from a more thorough discussion of the literature, a clearer presentation of the results (especially in the figure captions), and the inclusion of evidence to support some of the claims.

The Introduction was updated with more specific framing of prior literature (e.g., explicit mention of risk management, funding disparities, and diminishing marginal returns as the focus of prior work). New references were added throughout, including Sampat (2012) on mission-oriented NIH research, Ioannidis et al. (2019) on grant competition inefficiencies, Drummond et al. (2005) on health economic evaluation methods, and the Cassidy (2024) Senate report, throughout the introduction and discussion.

**Recommendations for the authors:**

**Reviewer #2 (Recommendations for the authors):**

The article would benefit from a more detailed analysis/discussion about the recovery of indirect costs for extramural research.

I note that the authors are from the University of Wisconsin, which is part of the IRIS network (https://iris.isr.umich.edu/iris-members-map/). They could work with IRIS (also called UMETRICS) to get a better sense as to the true costs of extramural research for each project (e.g., all labor costs, all equipment costs). The IRIS data are extraordinarily robust. Here's an example of an IRIS / UMETRICS paper: https://www.science.org/doi/10.1126/sciadv.abb7348.

They could, for example, re-do the analyses assuming that the recorded indirect cost covers only 70% of the true indirect costs. Thus, if they get $700,000 indirect costs from RePORTER, they should assume that the true indirect costs were $1,000,000. Similarly, they can add the costs of the time the PI spent writing the grant proposal, using the Bergstrom paper as a guide.

Another option would be to conduct sensitivity analyses taking into account ~30% incomplete indirect cost recovery (see here) and lost efficiency due to excess time writing grant proposals (see here).

We conducted a sensitivity analysis as requested inflating extramural indirect costs by 30%, citing the Droegemeier (2017) Congressional testimony as the basis for this estimate. The cost of grant-writing time is now acknowledged in the Discussion as an unreimbursed hidden cost of the extramural system, citing Ioannidis et al. (2019). This narrowed the gap between extramural research and intramural research, but did not close it completely. In addition, our updated regression (Supplemental Figure 4) showed similar trends as our main Figure 4, but with the Intramural advantage heightened and the Extramural advantage diminished. Both remained significant. We have also added to the discussion that there are additional costs and benefits that may not be fully captured in an analysis such as ours.

The authors appear to have used an agency-perspective for their cost-effectiveness analyses. Generally, it is preferable to use a wider societal perspective. While that may be difficult, the article would benefit from some discussion from the perspective of the government and universities.

We added a new paragraph explicitly acknowledging the agency-centered perspective and its limitations, noting that it does not capture the full economic cost borne by universities (startup costs, philanthropy, endowments, state contributions, graduate student training, faculty retention, infrastructure). The extramural program's contribution to the US workforce pipeline is specifically highlighted as a societal benefit not captured by the cost-effectiveness metrics.

**Reviewer #3 (Recommendations for the authors):**

Line 84-87: "The overrepresentation of viral research is likely because of the outsize investment toward the intramural Vaccine Research Center, and the cancer/genetics overrepresentation due in part because National Cancer Institute intramural investigators conduct research at that institute as well as at the NIH Clinical Center for their human genetics work." What evidence is there to support this claim?

A citation to the NCI Center for Cancer Research website was added to support the claim about NCI intramural investigators working at the Clinical Center and Center for Cancer Research, where vaccine research is extensively discussed.

Lines 107-109. "Given that NIH funding for intramural research has remained relatively constant as a percent of total funding over the years, this indicates larger single awards for intramural research while extramural investigators may increasingly require multiple concurrent grants to sustain their labs." Authors may consider adding a panel to Figure 2 showing the percentage of total funding of intramural vs. extramural funding.

Rather than adding a panel to Figure 2, we added a new Supplemental Figure 3 showing the cost breakdown and intramural percentage of total funding by year.

Discussion section: Are any of the findings of this study relevant to other funding agencies in the US (such as the National Science Foundation, the Department of Energy, and the Department of Defense)?

A new paragraph to the Discussion was added discussing implications for the Department of Defense (including the Congressionally Directed Medical Research Programs), NSF FFRDCs, and the Department of Energy's national labs and FFRDCs, arguing that the incentive-alignment logic likely generalizes across agencies.

Methods section: Please add an explanation of the technique used for propensity score matching.

A detailed step-by-step description of the PSM procedure was added, covering propensity score estimation, within-year matching, matched cohort construction, outcome regression on matched data, and visualization of results.

Figure 1: Please clarify if the relative ratio of intramural projects is calculated from the numbers of grants (as suggested in lines 95-96 and 98-100) or the numbers of publications (as suggested in lines 82-83 and 97-98).

Also, this figure would be more intuitive if, for each topic, it showed the relevant intramural number (as it currently does) and also the relevant extramural number.

The caption and Methods were updated to clarify that clustering and ratio calculation are based on projects/grants, not publications. A formula was added to the Methods to make the ratio calculation explicit. The figure itself was not modified to add extramural bars, though the ratio calculation already implicitly encodes both.

Figure 2: Please change "(red)" to "(blue)" in the caption, and remove the A as there is only one panel in this figure

Figure 4: Please change "(red)" to "(blue)" in the caption.

These changes have been made.

Lines 19-21: I suggest rewriting this sentence as follows:

"We find that extramural awards are more cost-effective for producing outputs commonly used for academic evaluation, such as publications and citations per dollar, while intramural awards are more cost-effective for generating research that influences future clinical work, more closely in line with agency's health goals."

The sentence was rewritten substantially in line with the reviewer's suggestion, now reading more clearly with "per dollar" removed as a parenthetical and the structure of the comparison clarified.

Lines 31-34: Please rewrite this sentence along the following lines to provide more context on previous research into the grant funding system:

Certain aspects of the grant funding system have been the focus of research, such as AAAA (Azoulay et al., 2009), BBBB (Goldstein and Kearney, 2020), CCC (Hoppe et al., 2019), DDDD (Lauer et al., 2017), EEEE (Wahls, 2018a) and FFFF (Wahls, 2018b), but the relative merits of intramural and extramural funding have received little attention to date.

The sentence was rewritten to name specific contributions of each cited paper (e.g., risk management, funding disparities, diminishing marginal returns), replacing the generic list of citations.

Lines 41-44: Please explain "merit score" and please add a reference to an article or website that explains the review process at the NIH.

"Merit score" was revised to "percentile ranking of overall impact merit score" and a citation to the NIH CSR website ("What happens to your application during and after review?," 2025) was added.

Lines 53-54: Please change Intramural to intramural (two instances, and also in line 284), and Extramural to extramural.

"Intramural" and "Extramural" were corrected to lowercase throughout.

Line 65-67: This sentence ("Potential advantages of the intramural approach are that researchers in the NIH's own laboratories allow the NIH to hire researchers whose research agendas more closely align with its mission.") reads awkwardly. Please clarify.

The sentence was rewritten to read more clearly: "An advantage of the intramural approach are that NIH has the direct ability to hire scientists whose research closely aligns with agency goals, and researchers do not need to devote time and effort on preparing and submitting grant applications."

Line 95-97: Authors should consider including an equation to help explain the following sentence: "The relative ratio of intramural projects for each topic was calculated by taking a ratio of the proportions of total grants a topic represented in the intramural vs. extramural portfolios. A relative ratio >1 signifies a higher share of intramural project publications on that topic relative to their share across all topics."

A formula was added to the Methods defining the topic-level ratio calculation explicitly.

Line 143: The phrase "may reflect the extra attention intramural investigators are afforded" reads awkwardly - please reword.

Reworded to "may reflect the extra time intramural investigators save because they do not have teaching and grant writing responsibilities."

Lines 303-304: This sentence ("First, as the renewal of project contracts may alter the topic and arrangement of the projects, we dropped 70,297 projects with renewal records in our data.") reads awkwardly. Please clarify.

Reworded to "Since the scientific focus of a study may drift over time, we dropped 70,297 projects with renewal records in our data."

Line 378-379: Please specify the model of ChatGPT used.

Done.
